# Arsenic Thiolation and the Role of Sulfate-Reducing Bacteria from the Human Intestinal Tract

**DOI:** 10.1289/ehp.1307759

**Published:** 2014-05-09

**Authors:** Sergio S.C. DC.Rubin, Pradeep Alava, Ivar Zekker, Gijs Du Laing, Tom Van de Wiele

**Affiliations:** 1 Laboratorium voor Microbiële Ecologie en Technologie, Faculteit Bio-ingenieurswetenschappen, Universiteit Gent, Gent, Belgium; 2 Centro Nacional de Investigaciones Biotecnologicas (CNIB), Cochabamba, Bolivia; 3 U.S. Environmental Protection Agency, Research Triangle Park, North Carolina, USA; 4 Institute of Chemistry, University of Tartu, Tartu, Estonia; 5 Laboratory of Analytical and Applied Ecochemistry, Universiteit Gent, Gent, Belgium

## Abstract

Background: Arsenic (As) toxicity is primarily based on its chemical speciation. Although inorganic and methylated As species are well characterized in terms of metabolism and formation in the human body, the origin of thiolated methylarsenicals is still unclear.

Objectives: We sought to determine whether sulfate-reducing bacteria (SRB) from the human gut are actively involved in the thiolation of monomethylarsonic acid (MMA^V^).

Methods: We incubated human fecal and colon microbiota in a batch incubator and in a dynamic gut simulator with a dose of 0.5 mg MMA^V^ in the absence or presence of sodium molybdate, an SRB inhibitor. We monitored the conversion of MMA^V^ into monomethyl monothioarsonate (MMMTA^V^) and other As species by high-performance liquid chromatography coupled with inductively coupled plasma mass spectrometry analysis. We monitored the sulfate-reducing activity of the SRB by measuring hydrogen sulfide (H_2_S) production. We used molecular analysis to determine the dominant species of SRB responsible for As thiolation.

Results: In the absence of sodium molybdate, the SRB activity—primarily derived from *Desulfovibrio desulfuricans (piger)*—was specifically and proportionally correlated (*p* < 0.01) to MMA^V^ conversion into MMMTA^V^. Inactivating the SRB with molybdate did not result in MMA^V^ thiolation; however, we observed that the microbiota from a dynamic gut simulator were capable of demethylating 4% of the incubated MMA^V^ into arsenous acid (iAs^III^), the trivalent and more toxic form of arsenic acid (iAs^V^).

Conclusion: We found that SRB of human gastrointestinal origin, through their ability to produce H_2_S, were necessary and sufficient to induce As thiolation. The toxicological consequences of this microbial As speciation change are not yet clear. However, given the efficient epithelial absorption of thiolated methylarsenicals, we conclude that the gut microbiome—and SRB activity in particular—should be incorporated into toxicokinetic analysis carried out after As exposure.

Citation: DC.Rubin SS, Alava P, Zekker I, Du Laing G, Van de Wiele T. 2014. Arsenic thiolation and the role of sulfate-reducing bacteria from the human intestinal tract. Environ Health Perspect 122:817–822; http://dx.doi.org/10.1289/ehp.1307759

## Introduction

Arsenic (As), particularly inorganic arsenic acid (iAs^V^), is a ubiquitous contaminant, a nonthreshold class 1 carcinogen (Cantor and Lubin 2007; Mandal and Suzuki 2002). Global impacts of geogenic As increase the risk for elevated As exposure through the consumption of contaminated drinking water and food (Francesconi 2010; Sun et al. 2012). Although orally ingested and bioavailable, As was previously thought to be mainly biotransformed in the liver (Watanabe and Hirano 2012); however, the literature also suggests that As can be converted presystemically during gastrointestinal transit (Kubachka et al. 2009a; Rowland and Davies 1981; Van de Wiele et al. 2010). Presystemic As metabolism is defined as the occurrence of As speciation changes due to physicochemical, enzymatic, or microbial metabolic processes in the gut before intestinal absorption and eventual bioavailability. Given the fact that As toxicity is primarily determined by its speciation, incorporating presystemic speciation changes into the risk evaluation process is warranted.

Analyses of human urine after iAs^V^ exposure revealed sulfur-containing As metabolites such as monomethyl monothioarsonic acid (MMMTA^V^) and dimethyl monothioarsinic acid (DMMTA^V^) (Hansen et al. 2004; Raml et al. 2007). Sulfur-containing arsenicals have also been detected in the urine and feces of experimental animals (Conklin et al. 2006; Kubachka et al. 2009b), in water (Fisher et al. 2007), and in vegetables (Yathavakilla et al. 2008). In addition, thioarsenicals have been produced within the headspace of a reaction tube containing a human fecal slurry and arsenate (Diaz-Bone et al. 2009). Furthermore, significant As thiolation has been observed with *in vitro* digestion of iAs^V^ under gastric conditions and with human colon microbiota (Van de Wiele et al. 2010). More recently, Pinyayev et al. (2011) showed that arsenate can be converted into methyl- and thioarsenicals by the anaerobic microbiota of the mouse cecum. However, the microbial mechanism of thioarsenical formation is not well understood. Moreover, the toxicity profiles remain under discussion (Dopp et al. 2010).

Given the importance of sulfate reduction by sulfate-reducing bacteria (SRB) in the human colon (Ley et al. 2006; Marchesi 2011), previous studies hypothesized that the SRB community in the gut may play an important role in the thiolation of arsenicals (Conklin et al. 2006; Van de Wiele et al. 2010). In the present study, we investigated to what extent thiolation of methylarsonic acid relies on the presence and metabolic activity of SRB from the human gut. Our findings suggest an active involvement of sulfate-reducing activity toward the gastrointestinal formation of thiolated methylarsenicals.

## Materials and Methods

*Chemicals, media, and microbial cultures*. Degassed and ultrapure 18 mΩ water [double-distilled ionized water (DDI); Millipore, Bedford, MA, USA)] was used to prepare the chromatographic mobile phase and the standard stock solutions. American Chemical Society–grade ammonium nitrate and ammonium dihydrogen phosphate (Fisher Scientific, Pittsburgh, PA, USA) and technical-grade EDTA, tetrasodium salt dihydrate (Fisher Scientific, Fair Lawn, NJ, USA), were used in the chromatographic mobile phase. Certified stock solutions of monomethylarsonic acid (MMA^V^) and sodium arsenate (Na_2_HAsO_4_·7H_2_O) were purchased from Chem Service (West Chester, PA, USA) and sodium molybdate (Na_2_MoO_4_·2H_2_O) was purchased from Sigma-Aldrich (St. Louis, MO, USA). Molybdate is not considered to be a bactericidal agent. Rather, it is a bacteriostatic agent: This compound merely inhibits the metabolic activity, limiting SRB in their growth and production of hydrogen sulfide (H_2_S). MMA^V^ and iAs^V^ stock solutions were prepared in DDI water at 0.1 g As/L and stored at –4°C.

MMMTA^V^ was synthesized using a mixture of MMA^V^ and H_2_S solutions. In a 1-mL glass vial, 900 μL of a 40-μg As/mL MMA^V^ solution and 100 μL of a saturated H_2_S solution were combined. The mixture was left overnight on a mechanical shaker for thorough mixing. Progress of the reaction was verified by liquid chromatography coupled with inductively coupled plasma mass spectrometry (LC-ICP-MS). Molecular identity of the product was checked by LC-LTQ-XL-MS [LC coupled with linear ion trap MS] and MS/MS (Alava et al. 2012). The MMA^V^ and H_2_S solutions were made as described by Alava et al. (2012). Briefly, we prepared a 40-μg MMA^V^/mL solution by combining 60 μL of a 1,850-μg MMA^V^/mL solution and 2.94 mL of a 10% vol/vol formic acid solution. Preparation of the saturated H_2_S solution was conducted in a 100-mL round-bottom flask. One gram of iron(II)sulfide (Harshaw Scientific, Cleveland, OH, USA) was supplemented with 2 mL of hydrochloric acid along with 4 mL of DDI. The mixture started to bubble instantly, releasing H_2_S gas. The H_2_S was bubbled into 15 mL of DDI water until the effervescence in the round-bottom flask subsided, creating the saturated H_2_S solution.

The Simulator of the Human Intestinal Microbial Ecosystem (SHIME) is a dynamic multicompartment simulator of the human gastrointestinal tract, mimicking the digestive processes of the stomach, small intestine, and ascending, transverse, and descending colon. The model has been validated against human *in vivo* conditions both in terms of gut microbial composition and metabolic activity (i.e., short-chain fatty acid profile) (Molly et al. 1994; Possemiers et al. 2006). The nutritional medium for the SHIME was prepared as described by Boever et al. (2000) and enabled the microbial communities of the different colon compartments to adapt to the nutritional and physicochemical conditions that prevail in the ascending, transverse, and descending colon. Briefly, 1 L of SHIME medium contained 1 g arabinogalactan, 2 g pectin, 1 g xylan, 3 g starch, 0.4 g glucose, 3 g yeast extract, 1 g pepsin, 4 g mucin, and 0.5 g cystein, at pH 7.

Postgate medium C (Grossman and Postgate 1953) was used to enrich SRB. It consisted of 4.5 g sodium sulfate, 0.5 g potassium dihydrogen phosphate, 0.06 g magnesium sulfate, 1.0 g ammonium chloride, 0.06 g calcium chloride, 1 g yeast extract, 0.1 g ascorbic acid, 0.004g ferrous sulfate, 6 g sodium lactate, and 0.3 g sodium citrate at pH 7.5. Modified Postgate medium C with different sulfate concentrations was obtained using a 4-fold dilution series in the concentration range: 0.007, 0.032, 0.125, and 0.5 M of sodium sulfate.

A pure culture of *Desulfovibrio desulfuricans* LMG 7529 was purchased from the Belgian Co-ordinated Collections Of Micro-organisms (BCCM-LGM; http://bccm.belspo.be/index.php) and was grown in the recommended medium 104 (BCCM-LMG). This strain is equivalent to ATCC 29577 (http://www.atcc.org). *D. desulfuricans* is still considered *D. piger* (Castro et al. 2000).

*Batch incubations of enriched, nonenriched, and pure cultures*. A first set of experiments was used to check to what extent the *in vitro*–cultured gut microbiota from the human inoculum was capable of performing iAs^V^ biotransformation in a manner similar to some of our previous findings (Alava et al. 2012; Van de Wiele et al. 2010). Briefly, 2 mL of descending colon suspension from the SHIME (see below) was anaerobically incubated with 30 μg iAs^V^/L for 48 hr and then analyzed for its As speciation profile with HPLC-ICP-MS as detailed by Van de Wiele et al. (2010).

The second set of experiments was more specifically targeted at evaluating the potential of gut microbiota and SRB to thiolate MMA^V^. We anaerobically incubated 2 mL of nonenriched SRB descending colon suspension from the SHIME for 48 hr with 0.5 mg/L MMA^V^. To favor SRB enrichment, 2 mL was sampled from the SHIME descending colon suspension and anaerobically incubated for 48 hr with 0.5 mg/L MMA^V^ in 18 mL Postgate medium C. The contribution of a reference sulfate-reducing strain toward MMA^V^ (0.5 mg/L) thiolation was assessed by incubating 2 mL of a pure culture of *Desulfovibrio desulfuricans (piger)* in 18 mL of culture medium 104 (BCCM-LMG).

A third set of experiments was performed to test the interindividual variability in MMA^V^ thiolation by human fecal microbiota. Fecal microbiota from seven different human individuals with no history of antibiotic treatment in the 6 months before the study (De Weirdt et al. 2010) and descending colon samples from three different SHIMEs were separately incubated with 0.5 mg/L MMA^V^ in Postgate medium C.

All incubation experiments with enriched and nonenriched SRB SHIME descending colon samples, human fecal microbiota and with *D. desulfuricans (piger)* were performed in the absence or presence of sodium molybdate (20 mM), a specific SRB inhibitor. In addition, heat-sterilized (120°C) incubations were used as an abiotic control. Incubations were performed under anaerobic conditions by capping the serum bottles with butyl rubber stoppers that are impervious to oxygen and subsequently flushing the bottles with nitrogen gas for 25 min. Cultures were then incubated at 37°C on a rotary shaker (180 rpm) for 48 hr. Aliquots of 2 mL/analysis were collected at four time points—0, 6, 24, and 48 hr—to monitor SRB activity, As speciation changes, and the molecular analysis of the microbiota. This study was approved by Ghent University’s ethical committee and registered by Belgian authorities (no. B670201214538).

*Continuous incubations in a SHIME.* Although the former batch experiments were conducted under SRB-favoring conditions, we performed a SHIME run to verify whether MMA^V^ speciation changes, particularly thiolation, also occurred under more representative conditions for the human gut that do not favor SRB. Moreover, the SHIME reactor also allows addressing colon-region–specific differences in the MMA^V^ thiolation potential.

The treatment consisted of a daily supplementation of 0.5 mg MMA^V^/L during 4 days. 20 mM of sodium molybdate was added on the third and fourth days of the SHIME run to inhibit SRB activity. Aliquots of 2 mL/analysis were collected from the ascending, transverse, and descending colon in order to monitor the conversion of MMA^V^ as well as SRB activity.

*SRB activity analysis and sample preparation for speciation analysis*. SRB activity was monitored by measuring H_2_S production using an analytical kit for detection of sulfide (Hach, Loveland, CO, USA) in an automated spectrophotometer (Nanocolor 500D; Macherey-Nagel, Düren, Germany) in 1:1 and 1:2 dilutions with anoxic water. To preserve the samples for further As speciation analysis, all samples were flash frozen with liquid nitrogen upon incubation and subsequently stored at –80°C. Before analysis with HPLC-ICP-MS, the samples were thawed and dissolved with ammonium carbonate (20 mM, pH 9.0) to minimize any sulfur–oxygen exchange while awaiting analysis (Conklin et al. 2006). Upon complete thawing, the sample was vortexed and centrifuged for 10 min at 10,400 ×*g* with an Eppendorf 5810R centrifuge (Brinkman Instruments, Westburg, NY, USA) to separate soluble As species from insoluble As (e.g., As sorbed to microbial biomass). The supernatant was filtered through a Millex-LCR 0.45 μm filter (Millipore) with a Luer-Lok 10-mL syringe (Becton, Dickinson and Co., Franklin Lakes, NJ, USA).

*As speciation analysis by HPLC-ICP-MS*. As speciation changes, and especially the conversion of MMA^V^ into MMMTA^V^ and arsenous acid (As^III^), were monitored with HPLC-ICP-MS matching the retention time and by comparing fragmentation pattern of prepared MMMTA^V^ on electrospray ionization–MS/MS with previously published conditions (Van de Wiele et al. 2010), using the limits of detection and quantification for the different As species indicated in Supplemental Material, Table S1. Briefly, 2 mL of the supernatant of each incubated sample was filtered using a 0.45-μm syringe-type PVDF (polyvinylidene difluoride) membrane filter, and the filtrate was diluted into 25 mL using DDI water. This filtrate was analyzed for total As content using ICP-MS. The same filtrate was used for speciation analysis using HPLC and optimized instrumental parameters for ICP-MS (PerkinElmer, Sunnyvale, CA, USA). Filtrates were diluted with the mobile phase and injected into the HPLC. We considered the sum of the As species in the filtrate observed chromatographically to be the bioaccessible fraction. We measured total As concentration in the digest filtrates using ICP-OES (ICP–optical emission spectroscopy) according to Alava et al. (2012, 2013). The applicable detection limit was 0.5 μg/L.

*Molecular analysis*. We performed polymerase chain reaction and denaturing gradient gel electrophoresis (PCR-DGGE) to obtain a general profile of the microbial community, used qPCR (quantitative PCR) to quantify the SRB (see Supplemental Material, Table S2), and created a clone library to identify the most dominant SRB species in the enriched and nonenriched SRB incubation experiments, as well as Illumina (San Diego, CA, USA) sequencing of nonenriched fecal samples. DNA extraction was carried out using the UltraClean® DNA Isolation Kit following the manufacturer’s instructions (Mo Bio Laboratories Inc., Carlsbad, CA, USA). PCR-DGGE of the 16S rRNA genes for all bacteria were amplified by PCR using the Taq-Polymerase Kit (Fermentas Inc., Hanover, MD, USA) with the general bacterial primers P338F and P518R and a GC-clamp of 40 bp on the forward primer (Muyzer et al. 1993). DGGE was performed using the Bio-Rad D gene system (Bio-Rad, Hercules, CA, USA). Clustering was based on the densitometric curves according to the Pearson correlation using BioNumerics software (version 5.1; http://www.applied-maths.com). (For the clone library, see Supplemental Material, p. 2.) Briefly, the PCR amplification of 16S rRNA gene fragments was carried out with the universal primers 63F and 1378R and cloned into the pCR®-TOPO® Vector of the TOPO TA cloning kit (Invitrogen, Carlsbad, CA, USA). The qPCR specific for SRB’s target gene, *dsrB* (dissimilatory sulfite reductase subunit beta; the gene for the key enzyme in dissimilatory sulfate reduction and phylogenetic marker for identification of SRBs) was carried out as described by Vermeiren (2011), adapted from Spence et al. (2008) (See Supplemental Material, Figure S1).

*Statistical analysis and sequences*. Batch incubation experiments of more than four groups were conducted in triplicate and SHIME run in duplicates. All data were analyzed using SigmaPlot, version 12.0 (SYSTAT Software Inc., San Jose, CA, USA). A one-way analysis of variance (ANOVA) test was performed to investigate intergroup differences. Two-case groups were covered by a *t*-test. Statistical differences for ANOVA and *t*-tests were significant if *p* ≤ 0.05, and highly significant if *p* ≤ 0.01. The nucleotide sequences data of the clone library are available in the European Molecular Biology Laboratory–European Bioinformatics Institute public database EMBL-EBI (http://www.ebi.ac.uk/; accession numbers HG531812 to HG531931).

## Results

In our batch incubation experiments with SHIME descending colon microbiota, the human gut microbiota actively metabolized iAs^V^ (30 μg = 100%) (Table 1). Importantly, 7% MMMTA^V^ formation was observed upon 48 hr of incubation. In addition, iAs^V^ was reduced to As^III^ (18%), and further transformation toward monomethyl arsonous acid (MMA^III^) (6.6%), MMA^V^ (3.2%), and dimethylarsinic acid (DMA^V^) (54%) was noted. These data show that the *in vitro* cultured microbial community from the human inoculum in these experiments had the potency to actively metabolize iAs^V^ (Table 1).

**Table 1:** Metabolic potency of colon microbiota (percent toward iAs^V^ speciation from a single experiment

Time (hr)	As^V^	As^III^	MMA^V^	MMA^III^	MMMTA^V^	DMA^V^	DMA^III^
0	100.0^*a*^	—	—	—	—	—	—
5	5.1	32.1	3.9	—	—	50.1	—
8	4.9	31.2	3.7	3.7	1.2	59.8	—
24	6.7	18.1	5.6	4.7	3.4	64.5	—
48	6.1	18.2	3.2	6.6	7.0	54.3	—
Abbreviations: DMA^III^, dimethylarsinous acid; DMA^V^, dimethylarsinic acid; MMA^III^, monomethyl arsonous acid. ^***a***^As^V^ standard at high concentration of 30 μg (100%).							

**Figure 1:**
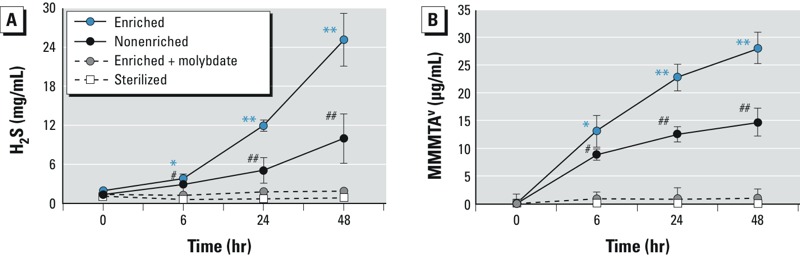
Sulfate-­reducing activity (H_2_S production) (*A*) and MMMTA^V^ formation (*B*) in enriched SRB (with and without sodium molybdate, the SRB inhibitor), non­enriched SRB, and abiotic (sterilized) cultures during 48 hr of incubation with MMA^V^ (0.5 mg/L). *p < 0.05, and **p < 0.01, by one-way ANOVA. ^#^*p* < 0.05, and ^##^*p* < 0.01, by two-case *t*-test based on non­enriched SRB (intermediary group).

Subsequently, we investigated to what extent SRB are involved in the thiolation of MMA^V^ toward MMMTA^V^. The batch incubations showed that SRB can be enriched with Postgate medium C and can be inhibited by the addition of sodium molybdate, and this was reflected in the sulfide production potential (Figure 1A). Molybdate is a well-known inhibitor of ATP-sulfurylase, thereby inhibiting SRB in their ability to produce sulfide but also limiting their ability to generate energy: Growth will therefore be reduced (Figure 1; see also Supplemental Material, Figure S1). Furthermore, we found that descending colon microbiota under enriched SRB conditions produced significantly more MMMTA^V^ (28 μg/L) than under nonenriched conditions (15 μg/L) (*p* < 0.01). In contrast, no MMA^V^ to MMMTA^V^ conversion was observed when the SRB-inhibitor sodium molybdate was supplemented (Figure 1B). In addition, both the incubations, with enriched and nonenriched SRB cultures, displayed a positive correlation (coefficient of determination) between the formation of MMMTA^V^ and production of H_2_S (*R*^2^ = 0.978 and *R*^2^ = 0.992, respectively). Finally, no speciation changes were observed in the abiotic control, in which heat-sterilized colon microbiota were incubated. However, the fraction of MMA^V^ remaining in the supernatant declined because of sorption to the dead organic biomass (see Supplemental Material, Table S3). This sorption shows the necessity of the contribution of sulfate-reducing activity to the thiolation process, whereas inactivation through a specific inhibitor or heat-sterilization removes the thiolation ability. It must be noted that the presence of H_2_S as such suffices to chemically produce MMMTA^V^ from MMA^V^ (see “Materials and Methods”). Hence, the As thiolation in the gut can be considered a chemical process that requires a biological trigger, that is, sulfide production by metabolically active SRB.

**Figure 2:**
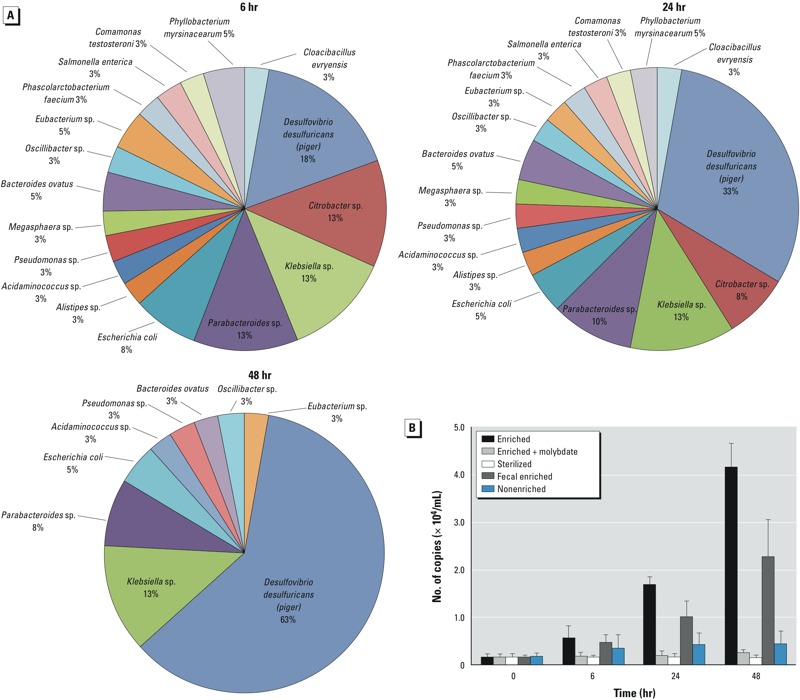
Molecular analysis of SRB cultures. (*A*) Clone library of 16S rRNA at the genus level (the operational taxonomic unit at 0.03%) during the enrichments of SRB culture in Postgate medium C. (*B*) Relative number of copies normalized to SRB *dsrB* gene of enriched, non­enriched, and pure fecal samples during 48-hr of incubation. Values are mean ± SD; *n* = 3.

To identify the dominant microbial species in enriched SRB cultures responsible for the thiolation of MMA^V^, we performed molecular analysis using the enriched and nonenriched SRB cultures. Analysis from the clone library revealed a dominant sequence over time (from 18% to 63% at 6 hr to 48 hr, respectively) with 99% of similarity to *D. desulfuricans (piger)* (Figure 2A). PCR-DGGE also showed an SRB-predominant band over the time observed in the enriched cultures (see Supplemental Material, Figures S2 and S3). Quantitative analysis with qPCR further confirmed the increasing abundance of SRB in the enriched cultures (Figure 2B). In addition, Illumina sequencing of nonenriched fecal incubations showed that *D. desulfuricans (piger)* is the most dominant SRB present in human gut (data not shown). Incubations of pure cultures of *D. desulfuricans (piger)* displayed sulfate-reducing activity and As-thiolation ability similar to that observed with the enriched SRB colon microbiota (see Supplemental Material, Figure S4).

**Figure 3:**
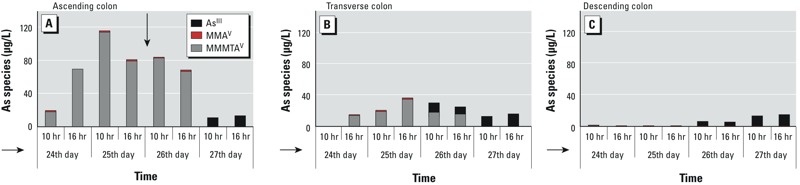
As speciation of MMA^V^ into MMMTA^V^ and As^III^ in the ascending (*A*), transverse (*B*), and descending (*C*) colon of the dynamic gut model SHIME. As speciation (i.e., thiolation of MMA^V^ into MMMTA^V^ or demethylation of MMA^V^ into As^III^), was measured daily during 4 days (the 24, 25, 26, 27th days) at two time points: 10 and 16 hr. The horizontal arrows indicate the sequence of colon compartments of the SHIME. The vertical arrow in (*A*) indicates the addition of the SRB inhibitor sodium molybdate.

Using SHIME as a dynamic simulator of the human gut (see Supplemental Material, Figure S5), we then investigated whether thiolation of 0.5-mg/L MMA^V^ is colon-region–specific under more representative conditions for the gastrointestinal tract (Figure 3). MMA^V^ thiolation was observed in the SHIME, with MMMTA^V^ formation primarily taking place in the ascending and transverse colon compartments at a rate of > 30 μg/L per day (Figure 3A,B). This resulted in high amounts of MMMTA^V^ (> 35 μg/L) in the ascending and transverse colon vessels, whereas only a minor amount was observed in the descending colon (Figure 3C). MMMTA^V^ formation took place within the first 10 hr upon supplementation of MMA^V^ (Figure 3). Adding sodium molybdate on the third and fourth days of the SHIME run to eliminate SRB activity did not result in a decrease in MMA^V^ conversion. Instead of MMMTA^V^ formation, demethylation of MMA^V^ occurred toward iAs^III^ (arsenous acid)—a process that primarily occurred in the distal colon regions (Figure 3C). (For information on the SHIME reactor distal colon regions and a scheme of As speciation, see Supplemental Material, Figures S5 and S6.)

**Figure 4:**
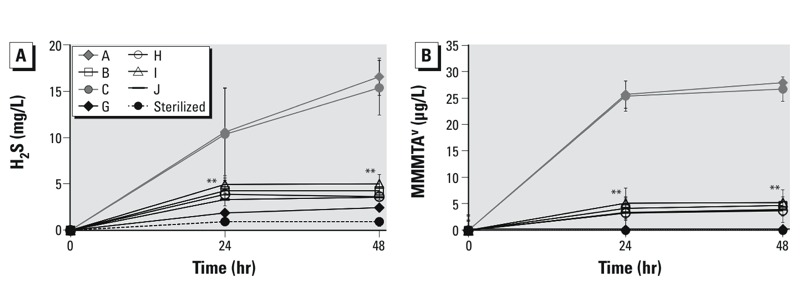
Interindividual variability of sulfate-­reducing activity (H_2_S production; *A*) and thiolation (MMMTAV formation; *B*) in different human fecal samples (from individuals A, B, C, G, H, I, and J) during 48 hr of incubation with MMA^V^. Abiotic controls are represented by the heat sterilized incubation of fecal microbiota from individual A. **p < 0.01, by one-way ANOVA and two-case *t*-test compared with the other groups.

Finally, we observed interindividual variability in the sulfate-reducing activity and MMA^V^ thiolation between different human fecal inocula (Figure 4). The fecal microbiota from individuals A and C displayed much higher levels of H_2_S (> 15 mg/L H_2_S) in comparison with the fecal microbiota from the other individuals (Figure 4A). This H_2_S production from fecal microbiota A and C corresponded with a pronounced production of MMMTA^V^ (> 20 μg/L) (Figure 4B). In contrast, for those fecal microbiota that displayed low SRB activity (around 5 mg/L H_2_S), only a limited amount of MMMTA^V^ was formed over time (around 4.5 μg/L at 24 and 48 hr). Moreover, the fecal microbial inoculum G displayed the lowest H_2_S production (< 2.5 mg/L) and no formation of MMMTA^V^. Overall, MMMTA^V^ formation and H_2_S production by fecal microbiota from each of the individuals were strongly correlated to one another (*R*^2^ = 0.994) after 48 hr.

## Discussion

The findings of the present study show that human colon microorganisms have the potency of presystemic As metabolism, similar to results previously obtained with rodent (Conklin et al. 2006; Kubachka et al. 2009a) and human gut microbiota (Van de Wiele et al. 2010). Moreover, this study shows that the active involvement of SRB from human origin contributes to the thiolation of MMA^V^ into MMMTA^V^. We observed this process both in enriched and nonenriched SRB in cultures of the descending colon, of human fecal microbiota, and of pure SRB isolates, as well as under more representative conditions for the human gut in the SHIME, a dynamic gut simulator. The active contribution of SRB was demonstrated by the high correlation between H_2_S production and MMMTA^V^ formation and by the lack of MMMTA^V^ formation and H_2_S production in the presence of the SRB inhibitor sodium molybdate. Moreover, our findings indicate that *D. desulfuricans (piger)* may be the principal microbe contributing to the As thiolation process. Although the metabolic activity of SRB has been well studied and even implicated in the methylation process of mercury (Gilmour et al. 2011), the role of metabolically active SRB, and particularly *D. desulfuricans (piger)*, toward As thiolation is a new finding.

Our observations parallel those of studies showing MMMTA^V^ formation upon incubation of iAs^V^ with human colon microbiota (Van de Wiele et al. 2010) or the formation of headspace thio-arsenicals when a human fecal slurry was incubated with arsenate (Diaz-Bone et al. 2009). Previous studies have reported that mouse cecal microbiota can trigger the formation of thioarsenosugars upon the incubation with arsenosugars (Conklin et al. 2006) as well as the production of methylated thioarsenicals from DMA^V^ by rat intestinal microbiota (Yoshida et al. 2001) and DMA^V^ conversion into trimethylarsine sulfide by mouse ceca (Kubachka et al. 2009a). Therefore, the presence of SRB in both the human and different animal gastrointestinal environments may be considered to be an important factor in the As thiolation process and, when considering the environmental presence of SRB, to also impact the biogeochemical cycles of sulfur and As (Muyzer and Stams 2008). Yet, As thiolation involves a chemical reaction that is biologically induced by metabolically active SRB. This view is supported by the possibility of chemically producing MMMTA^V^ by reacting MMA^V^ with a saturated H_2_S solution and corresponds with previous hypotheses that the presence of sulfide is sufficient to obtain interconversion between oxide and sulfide forms of MMA^V^, DMA^V^, and trimethylarsine oxide (Conklin et al. 2006).

Although the importance of As thiolation by endogenous SRB can be derived from the present data set, the results also demonstrate that the thiolation does not take place at the same rate throughout the entire gastrointestinal tract. In the present study, As thiolation appeared to be colon-region specific: Thiolation primarily occurred in the ascending and transverse colon. This observation is strongly supported by the fact that in the *in vivo* human colon, SRB are more abundant in the ascending and transverse colon, whereas homo-acetogens (which compete for reducing equivalents) are more abundant than SRB in the descending colon (Nava et al. 2012).

In addition, the inactivation of SRB activity by sodium molybdate in the SHIME colon compartments resulted in the demethylation of MMA^V^ toward iAs^III^ by descending colon microbiota. Although demethylation of MMA^V^ was previously reported for soil microbial communities (Yoshinaga et al. 2011), this is to our knowledge the first study to report As demethylation by human colon microorganisms. These findings are of toxicological concern. On the one hand, MMA^V^ demethylation is rather unexpected because iAs^III^ is more toxic than MMA^V^ (Naranmandura et al. 2011; Van de Wiele et al. 2010). On the other hand, the strongly reducing conditions that prevail in the SHIME colon compartments (–200 to –250 mV) may lead to the reduction of MMA^V^ toward its trivalent analogue, MMA^III^. The ability of this As species to generate highly reactive oxygen species and to induce DNA damage makes it an order of magnitude more toxic than iAs^III^ (Naranmandura et al. 2011). Although MMA^III^ was not detected under the dynamic incubation conditions of the SHIME, its production as intermediate is likely, as was also indicated by the finding of considerable amounts of MMA^III^ upon static incubation of iAs^V^ (Table 1). Supported by reports that MMMTA^V^ is several orders of magnitude less toxic than iAs^III^, and even less toxic than iAs^V^, we therefore consider intestinal MMA^V^ thiolation to be a detoxification reaction; however, further investigation is needed. Thiolated arsenicals display a highly variable toxicity profile. The monothiolated form of DMA^V^, DMMTA^V^—often found in the urine of iAs^V^-exposed individuals (Heitland and Köster 2008; Raml et al. 2007)—is one of the most toxic As species known, comparable to DMA^III^ (dimethylarsinous acid), whereas its dithiolated analogue, DMDTA^V^ (dimethyldithioarsinic), is almost harmless (Naranmandura et al. 2011). Whether SRB also contribute to the formation of DMMTA^V^, just as they do for MMMTA^V^, remains to be resolved.

Finally, As thiolation was characterized by a large interindividual variability. Again, the ability for a fecal microbiome to produce MMMTA^V^ correlated with the levels of H_2_S produced, further supporting the role for SRB as the basis of the thiolation process. Despite the enterotypes (Arumugam et al. 2011), the human gut microbiome is known for its high interindividual variability, which might also be reflected in the variable abundance of SRB in the colon lumen or on the colon mucosal surfaces (Nava et al. 2012). Although host genetic factors have been reported to contribute to the interindividual variability in As toxicity (Hernández and Marcos 2008), we propose that the gut microbiome must be incorporated as a factor that contributes to this variability.

## Conclusion

From the findings presented here, we conclude that gut microbiota of human origin can extensively metabolize As, with SRB being necessary and sufficient for the biologically induced thiolation of MMA^V^ into MMMTA^V^. The variability in the thiolation potency between different fecal inocula was reflected by a large interindividual variability in SRB abundance. In addition, eliminating SRB activity (as evidenced by H_2_S production) may also result in MMA^V^ demethylation to iAs^III^. Although the toxicological consequences of these microbial processes are not yet clear and the interindividual variability adds an extra layer of complexity over As toxicokinetics, our findings demonstrate the necessity to consider SRB and, by extension, the human gut microbiome when assessing risks from oral As exposure.

## Supplemental Material

(1.6 MB) PDF
