# Supplementary material for: Arsenic Thiolation and the Role of Sulfate-Reducing Bacteria from the Human Intestinal Tract
Source: Environ Health Perspect. 2014 May 9;122(8):817–22. doi: 10.1289/ehp.1307759 (PMC4123032; doi:10.1289/ehp.1307759)
Supplement: (1.6 MB) PDF [file ehp.1307759.s001.pdf]

Environmental Health Perspectives doi:10.1289/ehp.1307759

Supplemental Material

Arsenic Thiolation and the Role of Sulfate-Reducing Bacteria from the Human Intestinal Tract

Sergio S.C. DC.Rubin, Pradeep Alava, Ivar Zekker, Gijs Du Laing, and Tom Van de Wiele

Table of Contents **Page**

Materials and Methods 2

Molecular analysis 2

Clone library 2

qPCR 2

Table S1. Limits of detection and quantification for the different As species in SHIME matrix. 4

Table S2. qPCR measures and quantification of SRB 5

Table S3. Arsenic speciation in heat-sterilized colon microbiota incubation (ppb) 6

Figure S1. PCR products of *Desulfovibrio desulfuricans* dsrB gene of pCR-TOPO vector plasmid amplified with M13 primers and SRB enrich fecal sample with ET3 primers in 1.5% agarose stained with ethidium bromide 7

Figure S2. Enriched and non-enriched SRB growth curve. 8

Figure S3. PCR-DGGE of enriched SRB cultures during 48 hr 9

Figure S4. *Desulfovibrio desulfuricans (piger)* pure culture thiolation activity 10

Figure S5. The SHIME reactor 11

Figure S6. Scheme of arsenic speciation. Color arrows (bottom) represent the kind of As transformation 12

Materials and Methods

Molecular analysis

Clone library

For the clone library, the PCR amplification of 16S rRNA gene fragments was carried out with the universal primers 63F (5'-CAGGCCTAACACATGCAAGTC-3') and 1378r (5'-CGGTGTGTACAAGGCCCGGGAACG-3') using a T-100 thermal cycler (Biorad). PCR products were purified using GeneJet PCR purification kit (Thermo) and cloned into the pCR®-TOPO® Vector (Invitrogen, California) and transformed into DH5α *E.coli* chemical competent cells and grown on LB plates containing 50 μg/mL ampicillin, using beta-galactosidase screening. Forty positive clones of each time point (6h, 24h, 48h) were picked up for M13 pCR®-TOPO® PCR and automated sequencing (LGC genomics, Germany). Representative sequences were classified by comparison with BLASTN against the NCBI and EMBL database, to identify the best hit whose description did not correspond to an uncultured organism.

qPCR

Phenol-Chloroform DNA extraction of 1 ml of the sample was carry out of each time point (0, 6, 24, 48 hr) of the As incubations with human gut microbial cultures. Quantitative polymerase chain reaction (qPCR) to quantify sulfate reducing bacteria (SRB) through the functional DsrB gene was performed with the Rotor-Gene 6000 using SYBR Green PCR Master kit (Applied Biosystem). Template DNA (1 μl) was used in a reaction mixture containing 8.4 μL 2 × Quantitect SYBR Green PCR Mastermix, 0.3 μL (25 μM) of each forward and reverse ET3 set of *dsrB* primers ET3F1 (5'-AACAACATHGARTTYATGGT-3') and ET3R2 (5'-TAGCAGTTACCRCARTACAT-3') and H_2O to a final volume of 20 μL. Reaction conditions for quantification of DsrB were 95°C 10 min (1x), 94°C 30 sec, 50°C 30 sec and 60°C 50 sec (40x) followed by 72°C 10 min (x1). All samples were run in triplicate and H_2O replaced template in control reactions.

The standard curve to make a suitable standard for qPCR was calculated on the basis of a SRB enrich fecal dsrB PCR product (set primers ET3F1- ET3R2, size ≈ 200pb) (Figure 1S) purified using GeneJet PCR purification kit (Thermo), ligated and cloned into the pCR®-TOPO® Vector (Invitrogen, California) and transformed into DH5α *E.coli* chemical competent cells and grown on LB plates containing 50 µg/mL ampicillin, using beta-galactosidase screening. The size of the amplicon product from pCR-dsrB-TOPO using universal M13 forward (5′-GTAAAACGACGGCCAG-3′) and reverse (5′-CAGGAAACAGCTATGAC-3′) primers of the clone was ≈ 230 bp (Figure 1S). DNA sanger sequence showed 99% homology to dissimilatory sulfite reductase beta-subunit, dsrB of uncultured SRB (accession number HG932648.1) using the database searches (NCBI). The optimal DNA dilution was first tested and then used for the standard qPCR. Since SYBR green might also bind to nonspecific dsDNA, a melting curve was performed to assure the specificity of the PCR. By comparing the standard curve of pCR-dsrB-TOPO we determined the dsrB copy number/ml of the experimental sample. The determined average of the qPCR dsrB copy number/ml (triplicate) of each incubation is showed in Table S1.

Table S1. Limits of detection and quantification for the different As species in SHIME matrix.

Species	LOD (µg/L)	LOQ (µg/L)
As^{III}	0.40	1.2
DMA^{V}	0.30	0.9
MMA^{V}	0.40	1.2
As^{V}	0.40	1.2
$MMMTA^{V}$	0.30	0.9
MMA^{III}	0.30	0.9
DMA^{III}	0.60	1.8

Table S2. qPCR measures and quantification of SRB. The values represent number of copy/mL over the time. Data are presented as the mean of triplicate assays ± SD.

Time (hr)	Enriched SRB	Enriched SRB+ molybdate	Sterilized	Fecal	Non-enriched	*Desulfovibrio desulfuricans*
0	1703.79 ± 589	1687.95 ± 578	1503.79 ± 874	1603.56 ± 378	1689.85 ± 765	2003.32 ± 785
6	5686.74 ± 2501.34	1903.79 ± 765	1501.34 ± 498	4686.47 ± 1714.11	3345.34 ± 2987.32	5786.72 ± 2501.34
24	16978.3 ± 1498.25	2003.04 ± 987	1498.25 ± 827	9978.98 ± 3531.56	4098.12 ± 2585.25	17478.45 ± 1498.25
48	41642.3 ± 5075.32	2569.04 ± 687	1387.32 ± 567	22672.2 ± 7855.58	4254.55 ± 2853.98	45672.52 ± 5075.32

Table S3. Arsenic speciation in heat-sterilized colon microbiota incubation (ppb).

Time (hr)	As^{V}	As^{III}	MMA^{V}	DMA^{V}	$MMMTA^{V}$	MMA^{III}	DMA^{III}	Sum of species	Bioaccessible (%)
0	-	-	491.9	-	-	-	-	491.9	98.4
6	-	-	241.3	-	-	-	-	241.3	48.3
24	-	-	171.2	-	-	-	-	171.2	34.2
48	-	-	134.1	-	-	-	-	134.1	26.8

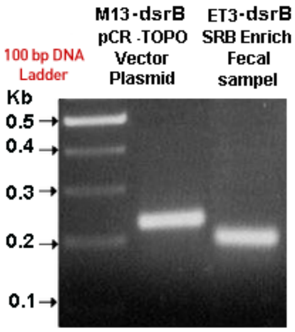

Figure S1. PCR products of *Desulfovibrio desulfuricans* dsrB gene of pCR-TOPO vector plasmid amplified with M13 primers and SRB enrich fecal sample with ET3 primers in 1.5% agarose stained with ethidium bromide.

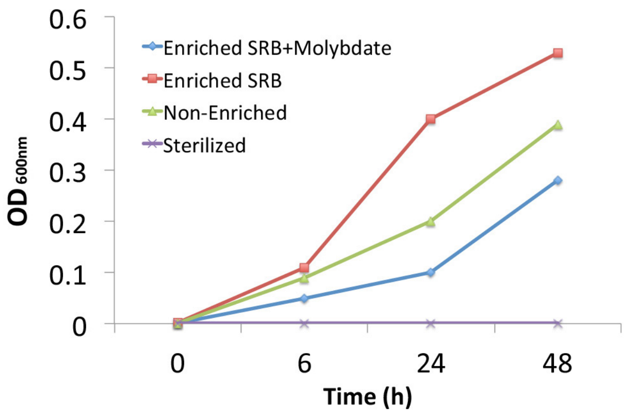

Figure S2. Enriched and non-enriched SRB growth curve. The growth curve of the experimental samples was carry out of each time point (0, 6, 24, 48 hr) from the different incubation conditions (in colors). The optical density (OD) of 1 mL of the experimental sample placed in a cuvette was measured at 600 nm in a Lange photometer. Observed values of optical measurement were plotted versus time to obtain a growth curve.

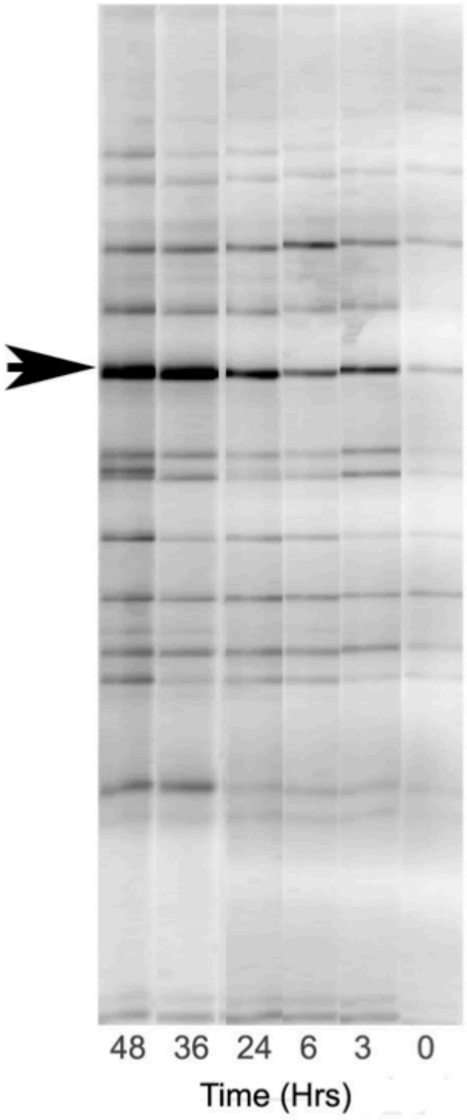

Figure S3. PCR-DGGE of enriched SRB cultures during 48 hr. The black arrow indicate the enrich band which correspond to *Desulfovibrio desulfuricans (piger)*.

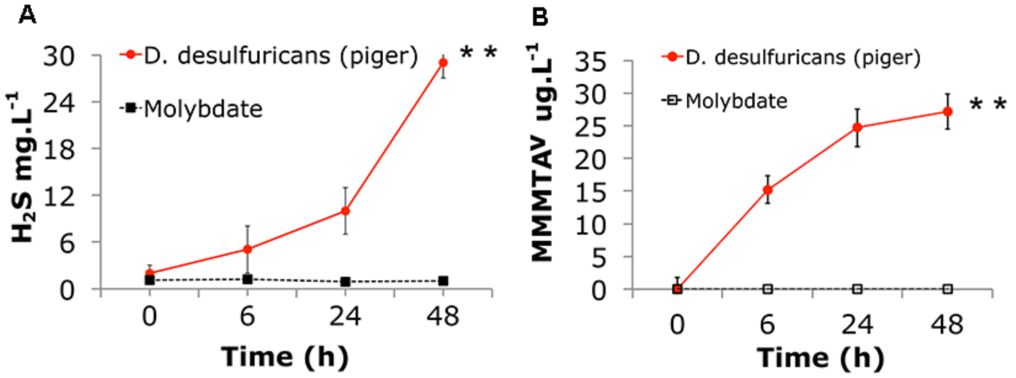

Figure S4. *Desulfovibrio desulfuricans (piger)* pure culture thiolation activity. A) *D. desulfuricans (piger)* pure culture activity (H_2S production) with and without inhibitor (Na-molybdate). B) $MMMTA^V$ formation induced by *D. desulfuricans (piger)* activity. Values are represented by mean ± SD, n=3. **$p < 0.01$.

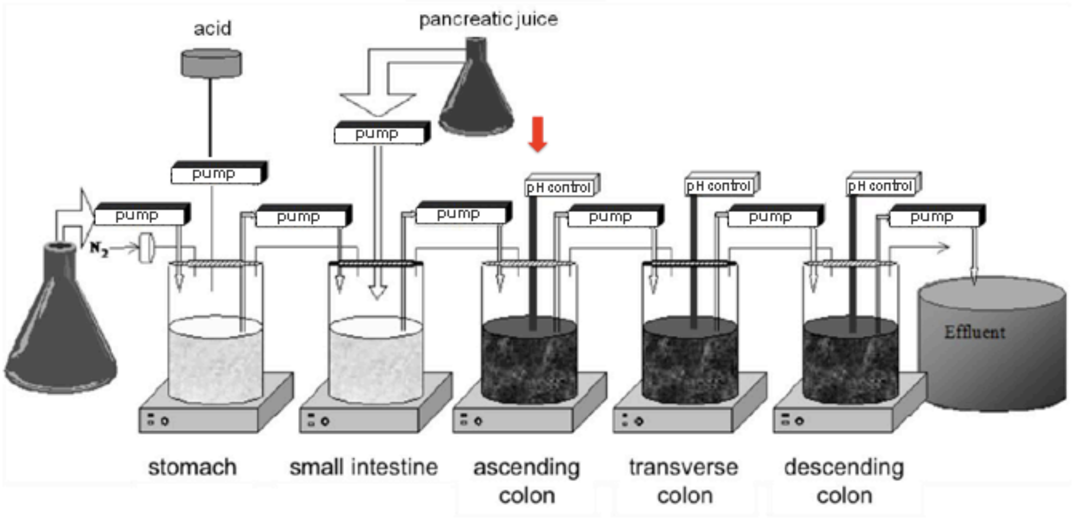

Figure S5. The SHIME reactor. The red arrow indicates the compartment that MMA^V and Na-molybdate were added during the dynamic experiment.

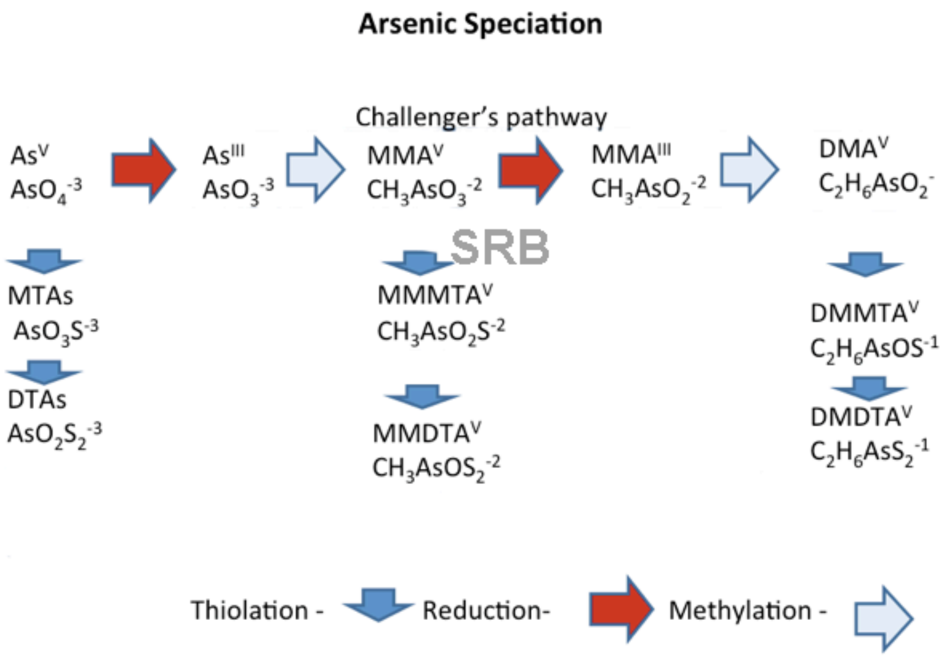

Figure S6. Scheme of arsenic speciation. Color arrows (bottom) represent the kind of As transformation. It is noted the role of the SRB in the thiolation of MMA^{V} into $MMMTA^{V}$.
